# A Combination of Caffeine Supplementation and Enriched Environment in an Alzheimer’s Disease Mouse Model

**DOI:** 10.3390/ijms24032155

**Published:** 2023-01-21

**Authors:** Martina Stazi, Silvia Zampar, Hans-Wolfgang Klafki, Thomas Meyer, Oliver Wirths

**Affiliations:** 1 Department of Psychiatry and Psychotherapy, University Medical Center (UMG), Georg-August-University, 37075 Göttingen, Germany; 2 Department of Psychosomatic Medicine, University Medical Center (UMG), Georg-August-University, 37075 Göttingen, Germany

**Keywords:** Alzheimer’ disease, amyloid-β, caffeine, physical activity, behavior, neuron loss, transgenic mice, Aβ4-42

## Abstract

A variety of factors has been associated with healthy brain aging, and epidemiological studies suggest that physical activity and nutritional supplements such as caffeine may reduce the risk of developing dementia and, in particular, Alzheimer’s disease (AD) in later life. Caffeine is known to act as a cognitive enhancer but has been also shown to positively affect exercise performance in endurance activities. We have previously observed that chronic oral caffeine supplementation and a treatment paradigm encompassing physical and cognitive stimulation by enriched environment (EE) housing can improve learning and memory performance and ameliorate hippocampal neuron loss in the Tg4-42 mouse model of AD. Here, we investigated whether these effects were synergistic. To that end, previous findings on individual treatments were complemented with unpublished, additional data and analyzed in depth by ANOVA followed by Bonferroni multiple comparison post tests. We further evaluated whether plasma neurofilament light chain levels reflect neuropathological and behavioral changes observed in the experimental groups. While a treatment combining physical activity and caffeine supplementation significantly improved learning and memory function compared to standard-housed vehicle-treated Tg4-42 in tasks such as the Morris water maze, no major additive effect outperforming the effects of the single interventions was observed.

## 1. Introduction

Caffeine is the most widely consumed psychostimulant drug worldwide [1], and a variety of epidemiological studies have linked caffeine intake with a decreased risk or incidence of common disorders such as diabetes mellitus [2] or cardiovascular [3] and neurodegenerative diseases [4,5]. It acts as an adenosine receptor antagonist, and a neuroprotective potential has been demonstrated in several preclinical studies in rodents when caffeine is consumed in moderate doses [6]. In mouse models of Alzheimer’s disease (AD), long-term oral caffeine supplementation resulted in a reduction of learning and memory deficits [7,8] as well as reduced β-amyloid deposition in cortex and hippocampus or lower β-amyloid (Aβ) plasma concentrations [9,10]. With regard to cognition in humans, several reports have shown that daily caffeine consumption equivalent to three or more cups of coffee may be neuroprotective and may thus slow down cognitive decline in aging individuals [11,12,13] and reduce the risk of developing AD later in life [14]. The latter finding has been recently confirmed in a large prospective cohort with more than 350,000 participants from the UK Biobank showing that drinking coffee and tea separately or in combination was associated with a lower risk of stroke and dementia [15]. Furthermore, individuals with mild cognitive impairment (MCI) who presented with high plasma caffeine levels were shown to be less likely to convert to AD in a case–control study [16]. In addition, a recent study employing [^11^C] Pittsburgh compound B-positron emission tomography (amyloid PET) reported a lower rate of Aβ brain positivity in non-demented older individuals with a coffee intake of two or more cups per day than in those with less coffee consumption [17].

In addition to the beneficial effects of nutritional factors, epidemiological studies also suggest an impact of cognitive stimulation [18] and physical activity with regard to delaying age-dependent decline of cognitive abilities and reducing dementia risk [19,20]. Regular moderate physical exercise is associated with increased cerebral blood flow [21], as well as larger hippocampal or gray matter volumes and better spatial memory in healthy seniors [22]. Data from the older Finnish Twin Cohort study showed that persistent vigorous leisure-time physical activity in adulthood protected from incipient dementia later in life [23]. The effects of physical activity have been mimicked in mouse models in a multitude of studies, with housing mice in an enriched environment (EE) conditions as the predominating paradigm [24,25]. This type of housing combines different cognitive parameters such as visual input or somatosensory stimulation with voluntary exercise, which is regarded as the main neurogenic and neurotrophic stimulus [26]. Beneficial effects of EE housing conditions in AD mouse models as well as wildtype (WT) mice on learning and memory outcomes have been reported in a multitude of studies [27,28,29,30]. While some studies show a concomitant reduction of Aβ pathology upon EE housing [31,32,33], unchanged Aβ levels [34,35,36] or even an exacerbation of extracellular amyloid plaque pathology [37] have been reported as well. We have previously analyzed the effects of long-term oral caffeine supplementation [7] or increased physical activity due to EE housing [38,39] in the Tg4-42 mouse model of AD. These mice overexpress the N-truncated Aβ4-42 peptide, which is among the most abundant Aβ variants found in amyloid plaques in human AD brains [40], and present with age-dependent CA1 neuron loss and associated behavioral impairments, but no overt amyloid plaque deposition [41]. We have shown previously that both EE housing and long-term oral caffeine treatment results in an amelioration of the Tg4-42 phenotype [7,39]. These data are used here as the baseline and were complemented with additional data from a combined treatment paradigm to investigate the potential synergistic effects of long-term oral caffeine supplementation and housing under EE conditions on behavioral and neuropathological outcomes in the Tg4-42 model.

## 2. Results

### 2.1. Weight Assessment in Tg4-42 Mice

**Figure 1:**
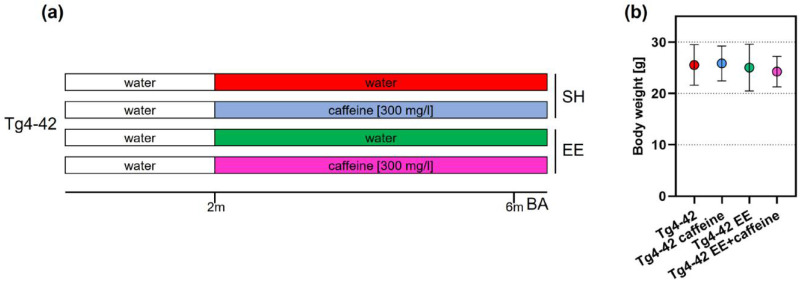
Experimental approach and weight assessment. After standard housing until the age of 2 months, Tg4-42 mice were assigned to standard (SH) or enriched housing (EE) conditions and received either tap water or caffeine-supplemented water for a period of 4 months. At 6 months of age, mice underwent a behavioral analysis (BA) with ongoing treatment (**a**). Body weight was assessed during the behavioral analysis and no statistically significant differences were detected among the treatment groups (**b**). One-way ANOVA followed by Bonferroni’s multiple comparison.

Tg4-42 mice were kept in standard (SH) or enriched housing (EE) conditions and received either tap water or caffeine supplementation for a period of 4 months (Figure 1a). No significant differences in body weight were observed between the different treatment conditions (Figure 1b).

### 2.2. Motor Performance in Tg4-42 Mice upon Enriched Environment Housing and Caffeine Treatment

**Figure 2:**
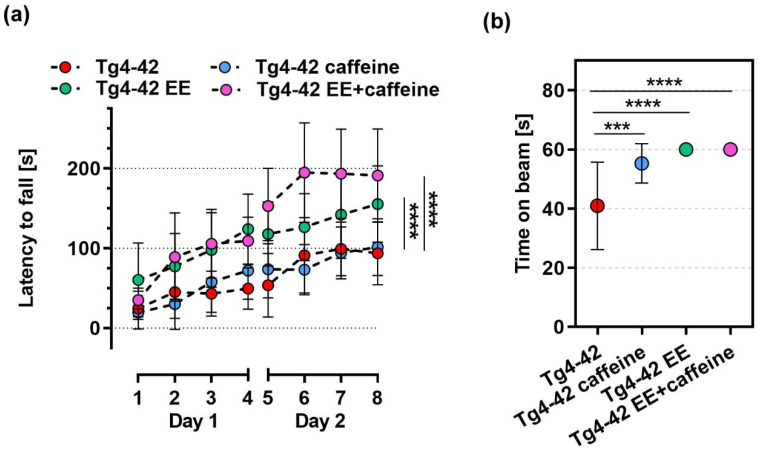
Effects of long-term EE housing and/or caffeine treatment on motor behavior in Tg4-42 mice. Both vehicle- and caffeine-treated EE mice showed a significantly improved motor performance in the rotarod task compared to the corresponding groups housed under SH conditions (**a**). In the balance beam task, Tg4-42 SH performed significantly worse compared to all other treatment groups (**b**). All data are given as mean ± SD. (**a**) Two-way repeated-measures ANOVA, followed by Bonferroni’s multiple comparison, (**b**) One-way ANOVA followed by Bonferroni’s multiple comparison: *** *p* < 0.001, **** *p* < 0.0001. Data from vehicle- and caffeine-treated SH and vehicle-treated EE Tg4-42 groups have been partially included in previous studies [7,39] and were part of a larger set of experiments in order to minimize experimental animal numbers.

Motor performances of 6 month-old Tg4-42 animals housed in standard or enriched environment conditions with either vehicle or caffeine supplementation were tested in the accelerating rotarod and balance beam tasks (Figure 2). The accelerating rotarod task is widely used to assess motor skill learning, motor coordination and balance. In general, all experimental groups improved their abilities to stay on the rotating rod over the course of the eight trials during the two days of testing (Figure 2a). As reported previously [7], caffeine-treated Tg4-42 mice housed under SH conditions showed a latency to fall that was indistinguishable from the vehicle-treated controls. An apparent treatment effect of caffeine on latency to fall in EE-housed mice did not reach statistical significance (*p* = 0.06, Two-way repeated measures ANOVA). However, when the individual time points were analyzed pairwise, caffeine-treated EE-housed mice performed statistically significantly better on day 6 (*p* < 0.001) and day 7 (*p* < 0.05, Two-way repeated measures ANOVA) compared to the EE-housed control group. Either vehicle- or caffeine-treated mice housed in an enriched environment showed significantly improved motor abilities compared to the respective standard-housed groups (*p* < 0.0001) (Figure 2a). In the balance beam task, caffeine-treated, standard-housed mice (*p* < 0.001) as well as vehicle- and caffeine-treated mice housed in an enriched environment (both *p* < 0.0001) outperformed vehicle-treated standard-housed Tg4-42 mice (Figure 2b).

### 2.3. Caffeine Treatment and/or Enriched Housing Improved Recognition Memory

**Figure 3:**
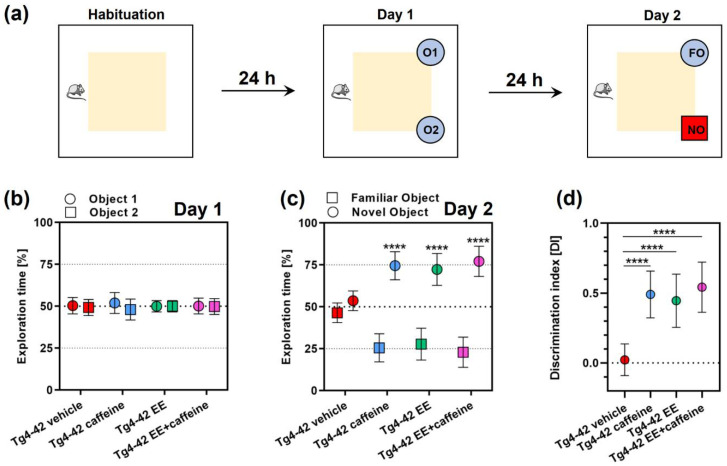
Impact of caffeine and EE housing on novel object recognition memory in Tg4-42 mice. Schematic illustration depicting the testing paradigm (**a**). The novel object recognition task (NOR) was used to assess recognition memory. All groups spent an equal amount of time with each of the identical objects on day one of the NOR (**b**), while during the testing phase on day two all treatment groups showed significant preference for the novel compared to the familiar object (**c**). Calculation of the discrimination index (DI) revealed that all treatment groups performed significantly better than untreated Tg4-42 mice (**d**). All data are expressed as mean ± SD. (**d**) One-way ANOVA followed by Bonferroni’s multiple comparison test, (**b**,**c**) Two-way ANOVA followed by Bonferroni’s multiple comparison test; **** *p* < 0.0001. Data from vehicle- and caffeine-treated SH and vehicle-treated EE Tg4-42 groups have been partially included in previous studies [7,39] and were part of a larger set of experiments in order to minimize experimental animal numbers.

Prior to the novel object recognition (NOR) task (Figure 3a), the open field task was carried out, representing also the habituation phase for this test. Tg4-42 mice housed under EE conditions spent significantly more time in the center region of the testing area compared to Tg4-42 as well as Tg4-42 caffeine-treated mice (*p* < 0.001 and *p* < 0.0001, respectively; Figure S1). No differences in exploration time were detected on day one of the NOR, with all groups spending an approximately equal amount of time with either of the two presented identical objects (Figure 3b). While all three treatment groups (i.e., SH + caffeine, EE + vehicle, EE + caffeine) spent significantly more time with the novel object on day two of the NOR (all *p* < 0.0001), no such difference was measured for the Tg4-42 control group (SH + vehicle), indicating disturbed recognition memory (Figure 3c) as shown before [7]. A calculation of the discrimination index revealed that all treatment groups showed significantly higher indices than Tg4-42 mice kept under SH conditions (all *p* < 0.0001), without any additive effects of enriched housing when combined with caffeine supplementation (Figure 3d).

### 2.4. Spatial Memory in Tg4-42 Mice upon Caffeine Treatment and Enriched Housing Conditions

Spatial reference memory was assessed using the Morris water maze (MWM) task. During the cued training, all analyzed groups showed a progressive decrease in their escape latencies, with the Tg4-42 EE group showing a significant decrease in latency compared to the Tg4-42 group (*p* < 0.05), albeit no difference in swimming speed was detected between the groups (Figure S2). A related finding was observed in the subsequent acquisition training, with Tg4-42 EE mice showing a significantly improved performance compared to the Tg4-42 (as shown before [39]) and Tg4-42 caffeine groups (both *p* < 0.05). Swimming speed was again unchanged among the experimental groups (Figure S2).

**Figure 4:**
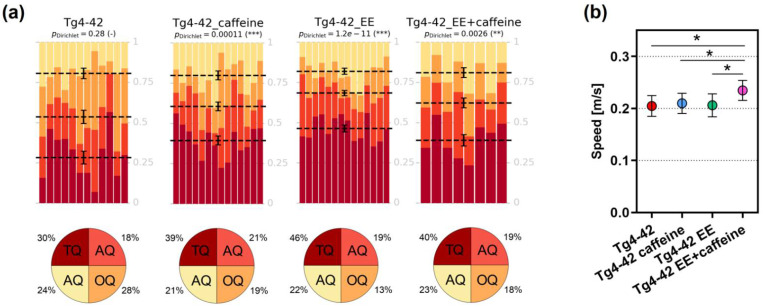
Spatial reference memory in Tg4-42 mice. (**a**) Except for Tg4-42, all treated groups displayed a clear and significant preference for the target compared to all the other quadrants, indicative of a rescued spatial reference memory. (**b**) Swimming speed during the probe trial showed a significant increase in Tg4-42 EE+caffeine compared to all other groups (all *p* < 0.05). Statistical analysis was performed using Dirichlet distributions (**a**) as described earlier [42]. Dirichlet *p*-values are indicated at the top of each heatmap. One-way ANOVA followed by Bonferroni’s multiple comparison test (**b**). * *p* < 0.05, ** *p* < 0.01, *** *p* < 0.001. All data are given as mean ± SD. TQ—target quadrant; AQ—adjacent quadrant; OQ—opposite quadrant. Data from vehicle- and caffeine-treated SH and vehicle-treated EE Tg4-42 groups have been partially included in previous studies [7,39] and were part of a larger set of experiments in order to minimize experimental animal numbers.

On the next day after the last day of the acquisition training, the probe trial was conducted without an escape platform. Except for the Tg4-42 control group (SH + vehicle), showing a more randomized presence in all quadrants, all treatment groups learned the task, as indicated by a significant preference for the target quadrant (Figure 4a). A comparison of the target quadrant occupancy revealed a significantly different increase only for the Tg4-42 EE versus Tg4-42 mice (one-way ANOVA followed by Bonferroni’s multiple comparison test; Tg4-42 vs. Tg4-42 EE, *p* < 0.01). An analysis of the swimming speed in the probe trial showed that mice in the Tg4-42 EE + caffeine group were significantly faster compared to all other groups (one-way ANOVA followed by Bonferroni’s multiple comparison test; all *p* < 0.05). Both Tg4-42 caffeine and Tg4-42 EE mice showed significantly more goal quadrant entries than Tg4-42 during the probe trial (*p* < 0.05 and *p* < 0.01, respectively). The same held true for the latency to the initial goal quadrant entry, which was significantly faster in Tg4-42 caffeine and Tg4-42 EE mice compared to the Tg4-42 control group (*p* < 0.01 and *p* < 0.001, respectively). Tg4-42 EE mice spent significantly more time in the goal quadrant than Tg4-42 mice (*p* < 0.01), while no differences in the number of platform entries, the platform time or the latency to the initial entry in the former platform position were detected (Figure S3).

### 2.5. CA1 Neuron Numbers in Caffeine and EE-Treated Tg4-42 Mice

**Figure 5:**
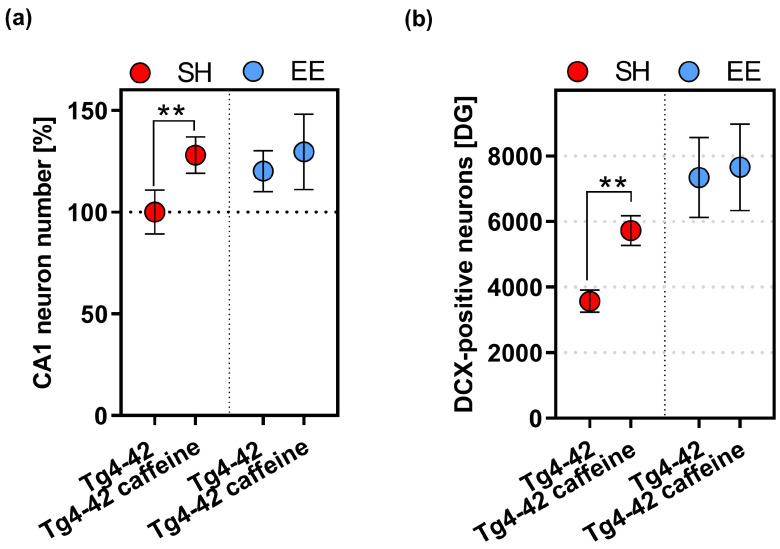
Quantification of CA1 neuron numbers and new-born neurons in the dentate gyrus (DG). (**a**) Caffeine treatment resulted in statistically significant higher CA1 neuron numbers in Tg4-42 mice housed under SH conditions, however, no effect of caffeine was detected under EE conditions. (**b**) Caffeine-treated Tg4-42 mice had increased numbers of DCX-positive neurons compared to vehicle-treated littermates. No additional effect of caffeine was observed in EE-housed mice (**b**). Two-way ANOVA followed by Bonferroni’s multiple comparison test (**a**,**b**). ** *p* < 0.01. All data are given as mean ± SD. Data from vehicle- and caffeine-treated SH and vehicle-treated EE Tg4-42 groups have been partially included in previous studies [7,39] and were part of a larger set of experiments in order to minimize experimental animal numbers.

As shown previously, homozygous Tg4–42 mice present with an age-dependent neuron loss in the CA1 region of the hippocampus [41,43]. This reaches a plateau at around 6 months of age [44], a time point when deficits become evident in a variety of behavioural tasks such as the NOR [43]. The pyramidal neuron number in the CA1 region was counted and calculated with the numbers of vehicle-treated Tg4-42 mice set to 100%. As shown before [7], caffeine treatment led to a significant amelioration of the neuron loss compared to vehicle-treated animals in standard housing conditions (*p* < 0.01). The same held true for the combined caffeine treatment and EE housing (*p* < 0.01). In contrast, EE-housed vehicle-treated Tg4-42 mice did not show a significantly altered neuron number compared to vehicle-treated SH mice. No additive beneficial effect of caffeine treatment was observed upon enriched housing (Figure 5a). A related finding was observed with regard to the number of new-born, doublecortin (DCX)-positive neurons in the dentate gyrus. Caffeine treatment resulted in a significant increase in neurogenesis rate compared to vehicle-treated animals in standard housing conditions (*p* < 0.01), and both vehicle- and caffeine-treated EE-housed mice showed significantly increased numbers compared to vehicle-treated SH mice (*p* < 0.0001, respectively) (Figure 5b).

### 2.6. Unchanged Neurofilament Light Chain Levels in the Plasma of Tg4-42 Mice

**Figure 6:**
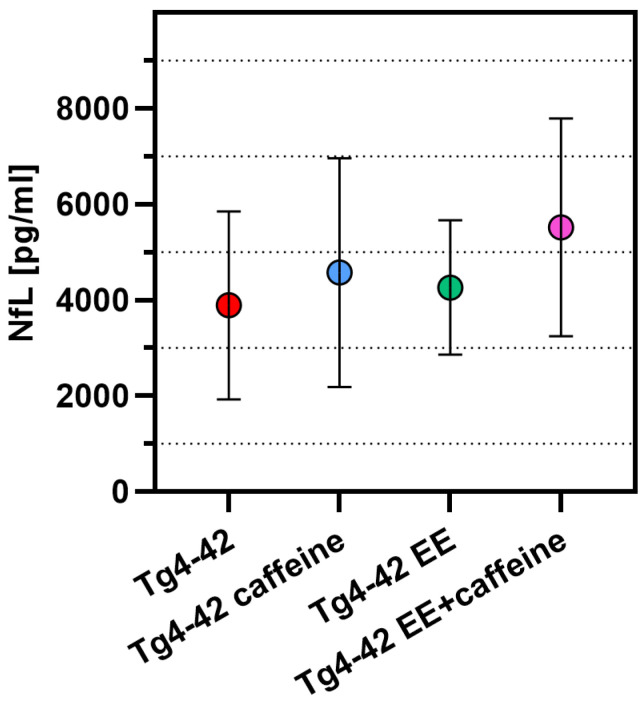
Neurofilament light chain (NfL) levels in plasma samples. No differences in NfL plasma levels, measured in pg/mL, were detected among the experimental groups. One-way ANOVA followed by Bonferroni’s multiple comparison test.

Plasma levels of cerebrospinal fluid (CSF)-derived neurofilament light chain (NfL) have been proposed as promising biomarkers reflecting neurodegeneration in AD or other related brain disorders. In order to evaluate whether plasma NfL levels may be a marker that reflects potential treatment effects in a preclinical setting, plasma NfL was measured in Tg4-42 mice housed in SH or EE conditions with or without additional caffeine supplementation. To control for potential matrix effects and evaluate the suitability of the assay for murine samples, pooled plasma samples from 6 month-old WT and a separate cohort of untreated Tg4-42 mice were measured in duplicate after two-, four-, six- or eight-fold dilution. The plasma NfL concentrations were back-calculated and plotted against the dilution factor for each sample. The back-calculated concentrations did not vary substantially between the different dilutions, suggesting that so-called matrix effects by interfering substances are negligible (Figure S4). We selected a six-fold dilution for all subsequent measurements. Compared to age-matched WT control mice, untreated Tg4-42 mice showed a very large increase in plasma NfL levels (342 ± 211 pg/mL vs. 4241 ± 2984 pg/mL, *p* < 0.05, Figure S4). High levels were also measured for all transgenic groups compared in the present study (Tg4-42 vehicle (3892 ± 1961 pg/mL), Tg4-42 caffeine (4577 ± 2386 pg/mL), Tg4-42 EE (4265 ± 1402 pg/mL) and Tg4-42 EE+caffeine (5519 ± 2273 pg/mL)), which were not statistically different among the vehicle or treatment groups (One-way ANOVA, *p* > 0.05) (Figure 6).

## 3. Discussion

In recent years, beneficial effects in terms of neuropathological or behavioral alterations as a result of enriched housing conditions or caffeine supplementation have been reported in a variety of studies employing transgenic mouse models [8,32,45,46]. As synergistic effects have been observed to a certain extent in WT mice [47], we analyzed whether a combined treatment paradigm might also result in an additive improvement in the Tg4-42 mouse model of AD. Mice were housed in either SH or EE conditions and received tap water or water supplemented with caffeine in a concentration of 300 mg/L. This dosage corresponds to ~five cups of coffee per day in humans [8,9,45] and has been demonstrated to result in considerable brain and plasma caffeine levels [8].

Tg4-42 mice at the age of 6–7 months show an impairment in motor learning and performance as demonstrated in the rotarod task [48]. While mice receiving only caffeine were indistinguishable from vehicle-treated SH mice in the rotarod task, mice housed in EE conditions showed a significantly improved motor performance. When housed in EE conditions, Tg4-42 mice receiving caffeine seemed to perform better than vehicle-treated mice, however, this did not reach statistical significance. In the balance beam task, all treatment groups showed a better performance in comparison to vehicle-treated SH Tg4-42 mice. EE-housed mice that were treated with caffeine showed a complete rescue of the phenotype and performance at WT levels, as well as Tg4-42 EE as shown in a previous study employing a part of this data set [39].

The beneficial effects of caffeine intake and increased physical activity or enriched housing on memory outcomes have been repeatedly described in preclinical rodent models of dementia [8,27,34,46,49,50]. Here we assessed potential additive effects of both treatments on recognition memory with the novel object recognition task, an established paradigm for hippocampus-dependent object recognition memory [51]. EE housing [39], as well as long-term oral caffeine supplementation [7] can rescue impaired object recognition at day 2 of the NOR. As shown here, the same held true for the combined treatment, however, without showing any further effect beyond the isolated treatment procedures. In a recent study applying the same experimental setup in C57Bl/6 WT mice, a significantly increased discrimination index in the combined treatment group compared to the other experimental conditions was observed [47]. Spatial reference memory was analyzed with the well-established Morris water maze paradigm [52], a task that is widely used to monitor learning and memory performance in rodent disease models. In our current study, analysis of target quadrant occupancy in the probe trial using Dirichlet distributions [42] indicated that, except for the Tg4-42 vehicle group, all groups learned the task. This was reflected in a significantly higher percentage of time spent in the target quadrant in comparison to the other three quadrants. Tg4-42 mice housed in an enriched environment showed the highest percentage of target quadrant occupancy among the groups, being significantly different from the Tg4-42 control group. Interestingly, this observation is in contrast to what has observed in a related study in WT mice, where mice in the combined treatment group showed a significantly higher target occupancy than vehicle-treated mice housed in either EE or SH conditions [47].

Caffeine and acute aerobic exercise on their own have been shown to significantly improve working memory accuracy in humans [53]. Several studies have investigated the potential synergistic effects of caffeine ingestion and exercise, mainly with regard to improvements in physical performance [54,55,56,57]. There are less data on cognitive outcomes, however, Hogervorst and colleagues demonstrated that an acute consumption of caffeine in the form of beverages or performance bars at the time of exercise significantly improved both endurance performance as well as complex cognitive abilities during and after strenuous [58] or exhaustive exercise [59], hinting at some effects of a combinational use, at least in healthy individuals. This is also reflected in a partial improvement in working memory in C57BL6/J mice undergoing the same experimental paradigm as in the present study [47]. Beneficial effects on hippocampal neurogenesis have been described for caffeine intake [7,60,61], as well as physical exercise or enriched environment housing [26,62,63]. On the contrary, a compromised proliferation of hippocampal progenitor cells has also been also reported after short-term caffeine treatment in a dose-dependent manner in in vitro experiments [64,65] or after a 4-week treatment in vivo [66]. Such a mechanism is unlikely in Tg4-42 mice undergoing long-term caffeine treatment as a significantly increased number of DCX-positive neurons was detected in mice housed in standard conditions [7]. This might be due to the more chronic treatment protocol applied. However, it is also obvious from the present analysis that physical activity is the most important stimulus, as vehicle-treated EE-housed Tg4-42 mice show a significantly higher number of newborn cells than Tg4-42 SH mice. There seems to be some kind of ceiling effect, as no further increase in newborn neurons was achieved by combining both treatments. The marker DCX has been demonstrated to allow for an accurate measurement of the rate of adult neurogenesis [67]. In addition to their role in adult neurogenesis, DCX-positive neural progenitor cells have been shown to be able to contribute to oligodendrocyte generation during remyelination in the adult hippocampus [68]. However, no evidence for increased de- and remyelination has been reported in the DG of Tg4-42 mice, suggesting that the observed increase in DCX-positive cells in the present study most likely represents an induction of neurogenesis.

We further investigated whether peripheral NfL levels, which have been considered as valuable surrogate markers of neurodegeneration [69], are altered in EE-housed and caffeine-treated mice. In good agreement with a previous study measuring NfL in CSF samples of Tg4-42 mice [70], significantly increased plasma levels were observed in Tg4-42 compared to age-matched WT animals. Though caffeine-treated Tg4-42 mice showed a significant amelioration of CA1 neuron loss compared to littermates housed under standard conditions, no statistically significant differences were measured among the different treatment groups. This might be due to the long half-life of this biomarker in blood, as it has been shown that NfL levels remained increased for months after traumatic brain injury [71].

## 4. Materials and Methods

### 4.1. Animals and Treatment

The generation of the Tg4-42 mouse model of AD has been described elsewhere [41]. In brief, this model utilizes the murine Thy1-promoter to drive the expression of a genetic construct consisting of the human Aβ4-42 peptide sequence, in the absence of the full-length amyloid precursor protein. This sequence was fused to the murine thyrotropin-releasing hormone (TRH) peptide to allow Aβ secretion [41]. Mice were generated and maintained on a C57Bl6/J genetic background. At 2 months of age, homozygous Tg4-42 mice were randomly assigned to either standard housing (SH) or EE conditions until the age of 6 months [39] and received standard chow and water. While mice in SH conditions were kept in standard laboratory cages (33 cm × 18 cm × 14 cm), larger rat cages (55 cm × 34 cm × 20 cm) equipped with running tunnels, plastic and metal wheels, nesting material, houses and toys were used in case of EE housing, which were cleaned and rearranged weekly to increase the sense of novelty, as done in previous studies [29,39]. Chronic oral caffeine treatment via drinking water was initiated at 2 months of age in further groups of animals housed in SH [7] and EE conditions (Figure 1a). Caffeine (Sigma-Aldrich, Schnelldorf, Germany) in a dosage of 300 mg/L, corresponding to ~5 cups of coffee per day in humans [9,45], was administered to the animals via drinking water and was maintained during behavioral testing, while vehicle-treated animals received tap water. Mice were housed in groups of 4–5 in all conditions to ensure social interactions; food and water were provided ad libitum. Data from vehicle- and caffeine-treated SH and vehicle-treated EE Tg4-42 groups have been partially included in previous studies [7,39] and were part of a larger set of experiments conducted in order to minimize experimental animal numbers. All animals were handled according to the German guidelines for animal care, and all experiments have been approved by the local animal care and use committee (Landesamt für Verbraucherschutz und Lebensmittelsicherheit (LAVES), Lower Saxony).

### 4.2. Behavioral Tasks

To assess potential beneficial effects of prolonged caffeine treatment and EE with regard to learning and motor behavior, mice were tested at 6 months of age at the end of the treatment period in a set of anxiety, motor and memory tests (SH-vehicle, n = 12; SH-caffeine, n = 14; EE-vehicle, n = 14; EE-caffeine, n = 8; Figure 1b). Animals were kept on a 12 h/12 h inverted dark/light cycle (light phase between 8 p.m. and 8 a.m.) and were sacrificed immediately after the last day of testing. All behavior experiments were carried out during the dark phase.

#### 4.2.1. Balance Beam

The balance beam task was used to assess balance and fine motor coordination as described before [7]. A 1-cm wooden beam is attached to two support columns 44 cm above a padded surface. At either end of the 50-cm long beam, a 9 cm × 15 cm escape platform is attached. Animal are placed on the center of the beam and released. Each animal is given 3 trials during a single day of testing, the time the animal remained on the beam is recorded and the resulting latencies to fall of all trials are averaged. The maximum time of 60 seconds is recorded if animals stay on the beam for the whole 60-second trial or escape to one of the platforms.

#### 4.2.2. Accelerating Rotarod

The accelerating rotarod test was used to evaluate motor learning, motor performance and balance abilities [72] (RotaRod, TSE Systems GmbH, Bad Homburg, Germany). The test was performed on 2 consecutive days with 4 trials per day and at least 15 min inter-trial intervals. Each mouse was individually placed on the rod, which accelerates from 4 to 40 revolutions per minute (rpm) over a maximal trial time of 300 s. The time spent on the rod was recorded as an indicator of motor performance (latency to fall [s]) and trials were terminated when animals fell off or the maximum time was reached. The apparatus was cleaned between trials with 70% ethanol to avoid odor cues.

#### 4.2.3. Open Field and Novel Object Recognition

Locomotor activity, exploratory behavior as well as anxiety levels were analyzed with the open field (OF) paradigm. Mice are allowed to freely explore a square arena (50 × 50 cm) during a single 5 min trial. Parameters such as total time spent in the central part of the arena, the total distance travelled, as well as the average speed were recorded using video-tracking software (ANY-maze, Stoelting Europe). Twenty-four hours after the OF, the novel object recognition test (NOR) was performed in the same arena, which was equipped with two identical objects (training phase). The NOR especially analyzes recognition memory and novelty preference and is a commonly used behavioral assay to test various aspects of learning and memory in rodents [73]. Mice were allowed to freely explore the objects for 5 min and were put back into their home-cage. Twenty-four hours later, one of the 2 objects was replaced with a novel one consistent in height and volume but different in shape and appearance (testing phase). Whenever the mouse sniffed the objects while looking at them, object exploration was scored, while climbing onto the object was not considered as exploration [74]. The percentage of exploration time for the novel object was calculated as follows:

$$=\frac{\left(time\;at\;novel\times 100\right)}{total\;exploration\;time}$$

In addition, observation scores were converted into discrimination indices (DI) to define novel versus familiar object exploration rates:

$$=\frac{\left(time\;at\;novel-time\;at\;familiar\right)}{total\;exploration\;time}$$

Odor cues were diminished by cleaning the arena as well as the objects with 70% ethanol in between the trials.

#### 4.2.4. Morris Water Maze

In order to assess spatial reference memory, the Morris water maze test (MWM) [52] was used as previously described [41]. The apparatus consisted of a circular pool (ø 110 cm) and a small escape platform (ø 10 cm). The pool was filled with opaque water in order to make the platform invisible and to facilitate the video tracking and mice were trained to learn to localize the position of the submerged platform. Initially, a 3 day “cued training” session was carried out (4 trials per day), in which the platform position was marked with a visible triangular flag. Next, proximal cues were added around the pool and 5 days of “acquisition training” (4 trials per day) were performed 24 h after the last trial of the cue training. The hidden platform remained stationary for each mouse in this training block with the triangular flag removed. After a period of 24 h after the last trial of the acquisition training, a “probe trial” was carried to assess spatial reference memory. During this final 60 sec trial, proximal and distal cues remained attached to the pool, while the platform was removed. Mice with successful spatial reference memory consolidation were expected to show a preference for the target quadrant, as the platform location was kept constant during the entire acquisition training phase. To prevent hypothermia, mice were kept under infra-red light to dry in between the trials. Swimming paths were recorded with video-tracking software (ANY-maze, Stoelting Europe) allowing the analysis of further parameters such as latency to first entry into the platform/target quadrant, time into the platform/target quadrant, entries into the platform/target quadrant, swimming speed, quadrant preference or latency.

### 4.3. Tissue Collection and Preservation

Mice were either euthanized by CO_2_ asphyxiation and subsequent cervical dislocation or were deeply anesthetized and transcardially perfused using ice-cold phosphate-buffered saline (PBS) before brains were carefully dissected. Post-fixation of the left hemisphere was carried out in 4% paraformaldehyde (PFA) in PBS for at least 24 h, before being transferred to a 30% sucrose solution (in PBS) for cryo-protection, while right brain hemispheres were post-fixed in 4% formalin solution at 4 °C for at least 72 h prior to embedding in paraffin (n = 5–6 per group).

### 4.4. Quantification of CA1 Neuron Number

Sagittal paraffin brain sections (bregma 1.08–1.32) of 4 µm thickness were cut and used for neuronal quantification in the hippocampal CA1 pyramidal cell layer as done previously [7]. In brief, after hematoxylin staining, neuronal nuclei were determined by their size and peculiar appearance clearly differing from glial cells (Figure S5). An Olympus BX-51 microscope equipped with a Moticam pro 282 camera (Motic, Germany) was used to acquire images of the CA1 area of the hippocampus at 400x magnification. The number of CA1 neurons per section (n = 3 per animal, 40 µm intersection distance) was counted using the manual cell counting tool implemented in ImageJ (version 1.52u, NIH), with the experimenter being blinded with regard to genotype and treatment throughout all the analysis. Data were normalized to Tg4-42 SH as the reference group (100%).

### 4.5. Analysis of Adult Neurogenesis

Series of coronal sections of 30 μm thickness were prepared from frozen cryo-protected brain hemispheres and every 10th coronal frozen section was stained with a free-floating staining protocol. Endogenous peroxidase activity was blocked by a 30 min immersion in 30% H_2_O_2_ in PBS following rehydration of a brain section series for 10 min with ice cold PBS. To ensure membrane permeabilization, sections were washed in PBS containing 0.01% Triton X-100 prior to an unspecific blocking step in PBS including 10% fetal calf serum (FCS) and 4% milk powder for 1 h at room temperature. Overnight incubation was carried out with a primary goat antibody against doublecortin (DCX, sc-8066, Santa Cruz Biotechnology, RRID:AB_2088494) that was applied in a 1:500 dilution in PBS containing 10% FCS. On the next day, sections were thoroughly washed with PBS incl. Triton X-100, followed by incubation with a secondary anti-goat biotinylated antibody (DAKO, Glostrup, Denmark) and visualization with the ABC method (Vectastain kit, Vector Laboratories, Burlingame, CA, USA) and using 3,3-diaminobenzidine as chromogen. The meander scan option of StereoInvestigator 7 (MicroBrightField, Williston, ND, USA) was used to count the total number of new-born neurons in the dentate gyrus (DG). To obtain the total number of new-born neurons per hemisphere, all DCX-positive cells counted in a given section (8–10 sections per animal) were multiplied by 10 [36]. The marker DCX has been demonstrated to allow for an accurate measurement of the rate of adult neurogenesis [67]. The experimenter was blinded with regard to genotype and treatment throughout the entire analysis. Only female mice were used (n = 5–6 per group) to quantify CA1 neuron numbers and adult neurogenesis in order to avoid possible bias due to sex-specific differences in brain size.

### 4.6. Neurofilament Light Chain Levels in Murine Plasma

For analysis of neurofilament light chain (NfL) in murine plasma, the R-PLEX human Neurofilament L assay (#F217X-3, MesoScale Discovery, Gaithersburg, MD, USA) employing MSD GOLD 96-well Small Spot Streptavidin plates (#L45SA-1) was used. In brief, calibrator peptide dilutions were prepared in Diluent-12, while antibody dilutions were prepared in Diluent-11 (MSD). Plasma samples were thawed on ice and measured after a 6-fold dilution with Diluent-12. For coating, plates were incubated at room temperature with continuous agitation for 60 min with 25 µL of biotinylated capture antibody diluted in Diluent-11. The plates were washed three times with wash buffer (PBS plus 0.05% Tween-20), prior to addition of 25 µL of the prepared calibrator peptide or sample dilutions per well and incubation for 60 min at room temperature with continuous shaking. After another three washing steps, 150 µL of MSD Gold Read buffer was added per well and electrochemiluminescent signals were recorded on a MESO QuickPlex SQ 120 instrument. Data were analyzed with the Discovery Workbench software package (MSD).

### 4.7. Statistical Analyses

Differences between groups were tested with one-way or two-way analysis of variance (ANOVA) followed by Bonferroni’s post-hoc test, as indicated. All data are given as means ± standard deviation (SD). Significance levels were given as follows: * *p* < 0.05, ** *p* < 0.01, *** *p* < 0.001; **** *p* < 0.0001. All calculations were performed using GraphPad Prism version 9.4.1 for Windows (Graph Pad Software, San Diego, CA, USA). MWM probe trial results were analyzed with Dirichlet distributions, as described previously [42] using the Dirichlet package from Eric Suh (Fitting the parameters of a Dirichlet distribution) available from: https://github.com/ericsuh/dirichlet (accessed on 23 June 2022). 

## 5. Conclusions

As it has been shown previously that a combination of EE housing and prolonged caffeine supplementation shows additive beneficial effects in WT mice to certain degree, we evaluated whether this is also true in an AD mouse model with neuron loss and learning and memory deficits. While either physical activity or caffeine treatment resulted in an amelioration of the behavioral phenotype and a restauration of impaired dentate gyrus neurogenesis, no further benefits of a combined treatment paradigm were observed, indicating a kind of ceiling effect in this disease model.

## Data Availability

Original data is available from the authors upon reasonable request.

## Supplementary Materials

The following are available online at https://www.mdpi.com/article/10.3390/ijms24032155/s1, Figure S1: Open Field, Figure S2: Water Maze—Training sessions, Figure S3: Water Maze—Probe trial parameters, Figure S4: Plasma NfL assay, Figure S5: Example images CA1 pyramidal layer.
