# Supplementary material for: A Combination of Caffeine Supplementation and Enriched Environment in an Alzheimer’s Disease Mouse Model
Source: Int J Mol Sci. 2023 Jan 21;24(3):2155. doi: 10.3390/ijms24032155 (PMC9916825; doi:10.3390/ijms24032155)
Supplement: Supplementary file 1 [file ijms-24-02155-s001.zip › ijms-2143600-supplementary.pdf]

Supplementary Material

A Combination of Caffeine Supplementation and Enriched Environment in an Alzheimer’s Disease Mouse Model

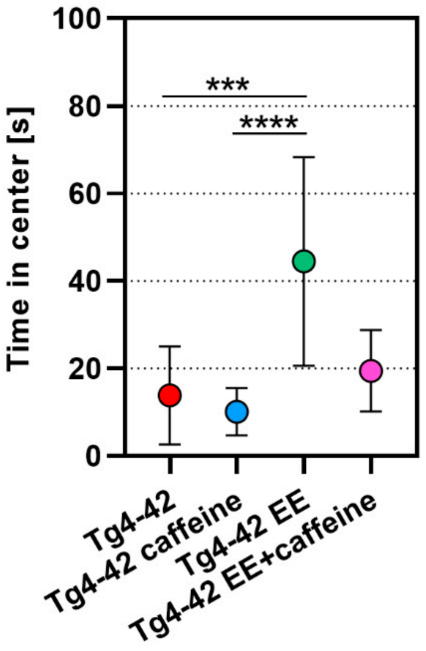

Figure S1. Tg4-42 mice housed under EE conditions spent significantly more time in the central area of the open field compared to Tg4-42 and Tg4-42 caffeine mice. One-way ANOVA followed by Bonferroni’s multiple comparison test. ***$p < 0.001$, ****$p < 0.0001$. All data are given as mean ± SD. Data from vehicle- and caffeine-treated SH and vehicle-treated EE Tg4-42 groups have been partially included in previous studies [1, 2] and were part of a larger set of experiments in order to minimize experimental animal numbers.

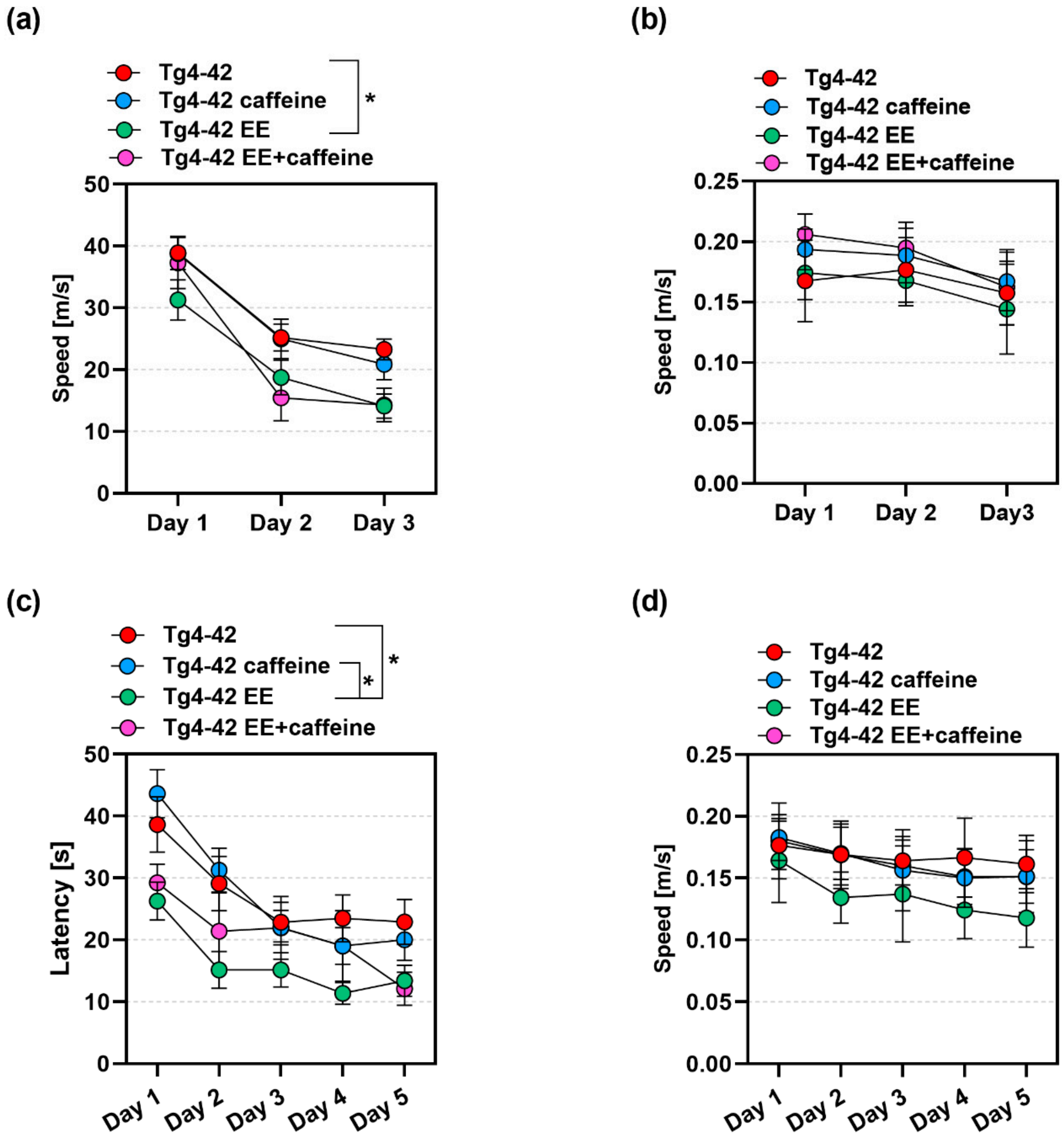

Figure S2. During the cued (**a**, **b**) and acquisition training phase (**c**, **d**), Tg4-42 mice housed under EE conditions mice displayed a reduced escape latency compared to Tg42-42 SH and Tg4-42 caffeine groups (**a**, **c**), while swimming speeds were unaltered (**b**, **d**). Two-way repeated measures ANOVA followed by Bonferroni's multiple comparison test. *$p < 0.05$. All data are given as mean ± SD. Data from vehicle- and caffeine-treated SH and vehicle-treated EE Tg4-42 groups have been partially included in previous studies [1, 2] and were part of a larger set of experiments in order to minimize experimental animal numbers.

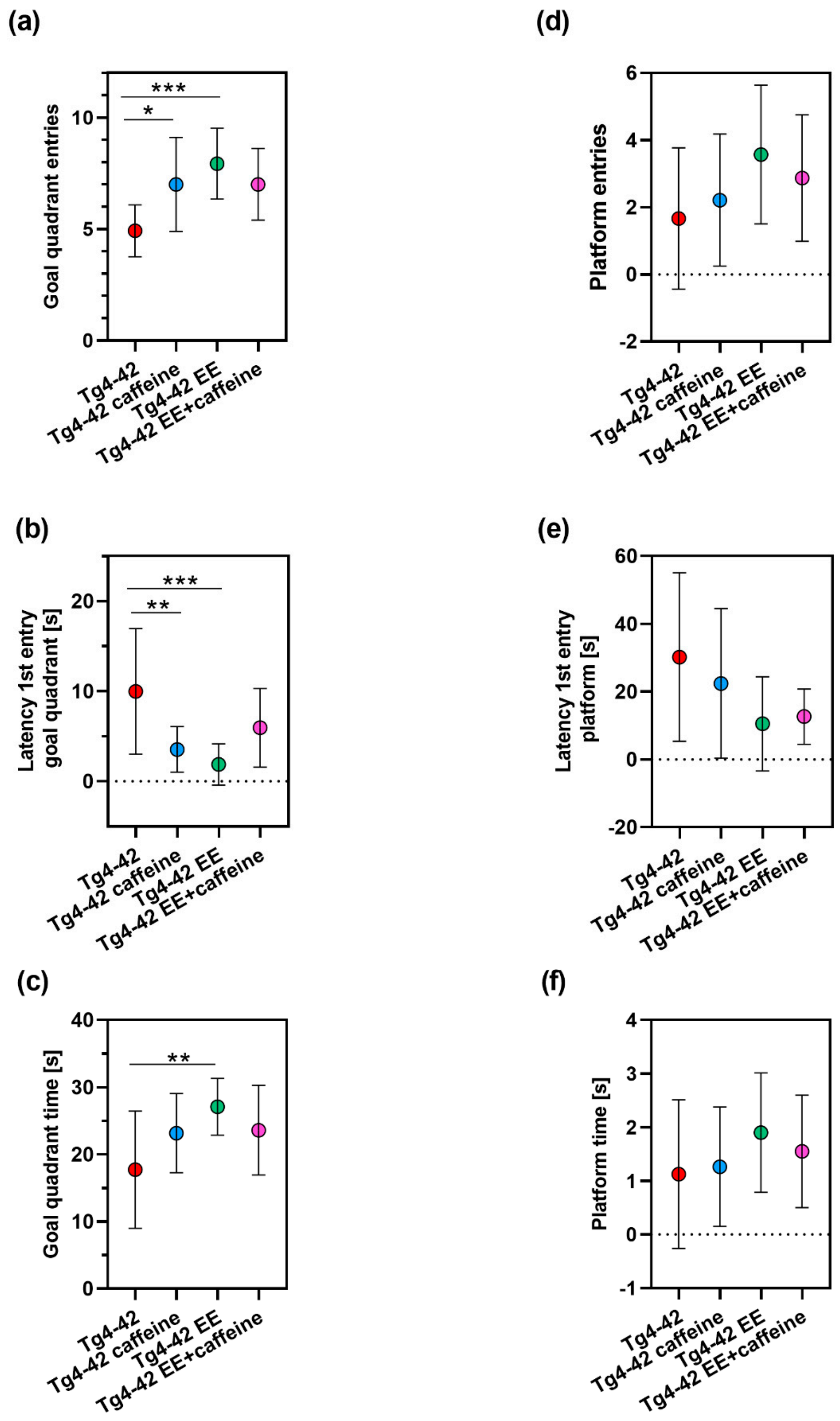

Figure S3. Compared to Tg4-42 mice, Tg4-42 caffeine and Tg4-42 EE groups showed more goal quadrant entries (**a**), as well as a significantly reduced latency for the initial goal quadrant entry (**b**). Tg4-42 EE mice spent significantly more time in the goal quadrant than Tg4-42 mice (**c**), while no statistically significant differences in the number of platform entries (**d**), the latency to the initial entry in the former platform position (**e**) or the platform time (**f**) were detected. One-way ANOVA followed by Bonferroni's multiple comparison test. *$p < 0.05$, **$p < 0.01$, ***$p < 0.001$. All data are given as mean ± SD. Data from vehicle- and caffeine-treated SH and vehicle-treated EE Tg4-42 groups have been partially included in previous studies [1, 2] and were part of a larger set of experiments in order to minimize experimental animal numbers.

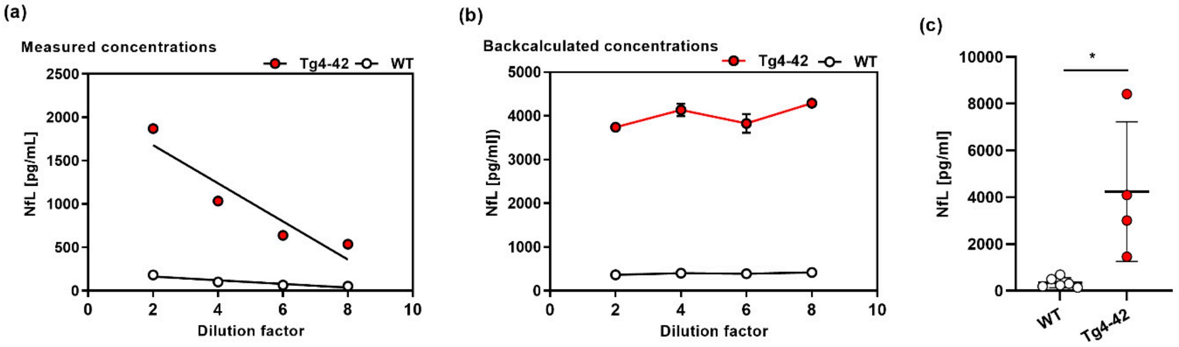

Figure S4. Pooled plasma samples from 6-month-old Tg4-42 and WT mice were measured in different dilutions (**a**). The plasma NfL concentrations were back-calculated and plotted against the dilution factor (**b**). Plasma samples of 6-month-old WT (n = 6) and Tg4-42 (n = 4) mice were measured in 6-fold dilution, revealing significantly increased NfL levels in Tg4-42 mice (**c**). **p* < 0.05, two-tailed unpaired t-test.

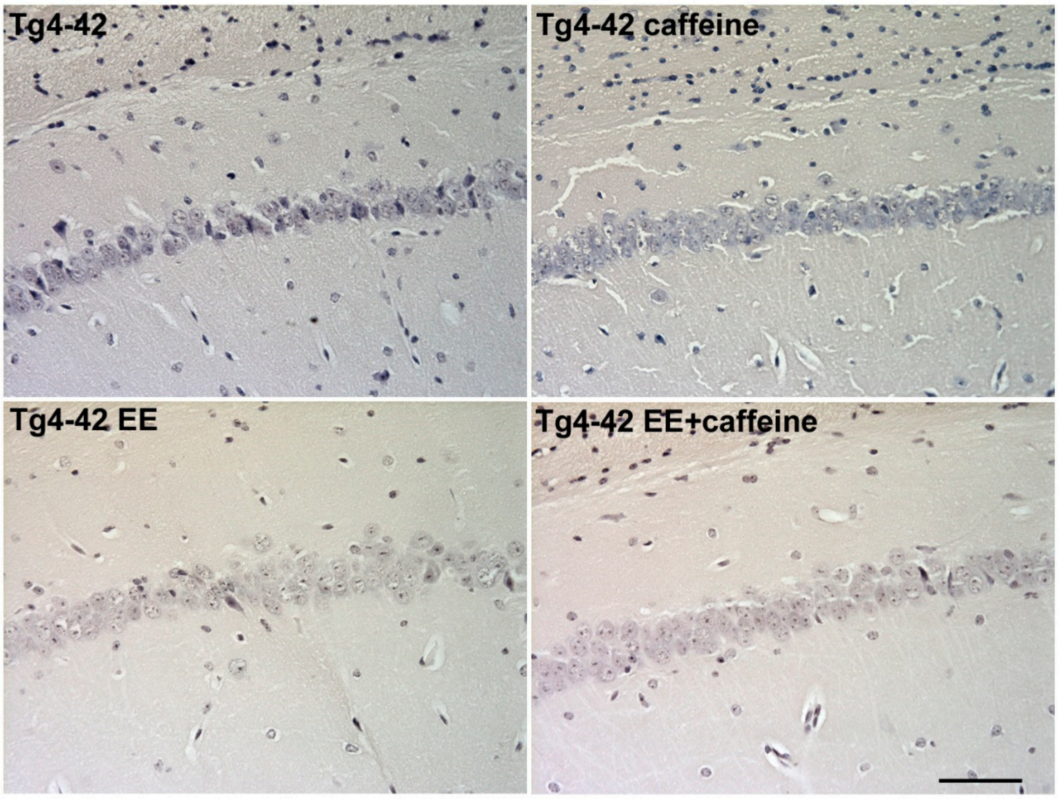

Figure S5. Example images of CA1 pyramidal neurons at 6 months of age. Sagittal brain sections were stained with hematoxylin and images were taken at 400x magnification. Scale bar: 50 µm.
